# Synthesis and Catalytic and Biological Activities of Silver and Copper Nanoparticles Using* Cassia occidentalis*

**DOI:** 10.1155/2018/6735426

**Published:** 2018-05-02

**Authors:** Manjul Gondwal, Geeta Joshi nee Pant

**Affiliations:** Department of Chemistry, Hemvati Nandan Bahuguna Garhwal University, Srinagar, Garhwal, Uttarakhand, India

## Abstract

An ecofriendly and green method for the synthesis of silver and copper nanoparticles has developed using aqueous leaves extract of* Cassia occidentalis*. The formation of AgNPs and CuNPs was monitored by measuring the UV-Vis spectra. The morphology and crystalline phase of the metal nanoparticles were determined using transmission electron microscopy (TEM) and scanning electron microscopy (SEM) with X-ray energy dispersive spectrophotometer (EDX) and X-ray diffraction (XRD). The synthesized metal nanoparticles were generally found to be spherical and oval in shape. The AgNPs and CuNPs showed highly potent antibacterial activity against* Escherichia coli* and* Salmonella typhi* bacteria, respectively. The CuNPs showed higher radical scavenging activity than AgNPs. The AgNPs showed lower haemolysis (1.7%) exhibiting lesser toxicity as compared to CuNPs. The CuNPs have better catalytic ability for the reduction of 4-nitrophenol and 2-nitrophenol as compared to AgNPs.

## 1. Introduction

Metal nanoparticles have been studied for their remarkable optical and electronic properties and their applications in the areas such as optics, optoelectronics, catalysis, nanostructure fabrication, and chemical sensing [1]. The physical and chemical methods used for the synthesis of metal nanoparticles are expensive and potentially dangerous to the environment [2]. These methods have many drawbacks including use of toxic solvents, generation of hazardous byproducts, and high energy consumption. So, there is a need to develop environmentally benign procedures for synthesis of metal nanoparticles.

Metal nanoparticles (MNPs) such as silver and copper nanoparticles have been known to have inhibitory and bactericidal effects [3]. The most widely used known applications of silver metal and silver nanoparticles are in the medical industry. These include topical ointments and creams containing silver to prevent infection of burns and open wounds [4]. Copper nanoparticles have been used as disinfectants in water treatment plants, food processing, wound healing ointments, bandages, and so on because of their antibacterial as well as antiviral properties [5, 6].

*Cassia occidentalis *Linn. (Caesalpiniaceae) is a native plant of southern India, Burma, Sri Lanka, and scattered from foothills of Himalayas to West Bengal. It is used against fever, menstrual problems, and tuberculosis, as diuretic, in anaemia, in liver complaints, and as a tonic against general weakness and illness [7].* C. occidentalis* leaf extracts exhibit antibacterial, antimalarial, anticarcinogenic, and hepatoprotective activity [8–10]. Chemical constituents isolated from* C. occidentalis* include sennoside, anthraquinone glycoside, fatty oils, flavonoid glycosides, galactomannan, polysaccharides, and tannins [11, 12].

Plant extract used in the green synthesis processes has received increasing attention for the development of metal nanoparticles [13]. The green synthesis techniques are generally synthetic routes that utilize relatively nontoxic chemicals to synthesize nanomaterials, and include the use of nontoxic solvents such as water, biological extracts, and microwave assisted synthesis. In the present paper, silver and copper nanoparticles were synthesized from leaves' extract of* C. occidentalis* and characterized by various spectroscopic techniques and examined for antibacterial, antioxidant activity and for toxicity. Further, these nanoparticles were employed as catalysts in the reduction of nitro compounds.

## 2. Experimental Section

### 2.1. Reagents and Materials

Silver nitrate (AgNO_3_), cupric nitrate (Cu(NO_3_)_2_), and nitrophenols were obtained from Merck Chemicals. All glassware has been properly washed with distilled water and dried in oven before use.

### 2.2. Green Synthesis of Silver Nanoparticles

Fresh leaves of* C. occidentalis* were collected from Garhwal University campus in the month of April. They were washed with distilled water and air-dried at room temperature. The 10 gm of leaves was kept in a beaker containing 100 ml deionized water and boiled for 30 min. The extract was cooled down and filtered with Whatman filter paper no. 1 and extract was stored in a refrigerator at 4°C.

The silver nanoparticles were synthesized by treating the freshly prepared 1 mM silver nitrate and stored under dark conditions with aqueous extract of the plant. The reaction mixture was prepared in a ratio of 9 : 1 (V/V) comprised of freshly prepared silver nitrate solution and plant extract, respectively [14]. The solution was stored at room temperature for 24 hours for the complete settlement of nanoparticles. After 24 hours the reaction mixture was centrifuged at 5000 rpm for 15 minutes and pellets were collected followed by washing with deionized water and dried in watch glass at room temperature. The resulting dried product was crushed into powder and stored in air tight container for further analysis.

### 2.3. Green Synthesis of Copper Nanoparticles

The 10 gm of leaves was boiled with 100 ml deionized water for 30 min. The extract was cooled down and filtered with Whatman filter paper no. 1 and extract was stored in a refrigerator at 4°C. The CuNPs were prepared by adding 10 ml of aqueous extract of plant material to 50 ml of 1 mM aqueous solution of cupric nitrate [15]. The mixture was irradiated in microwave oven for 4 hours and allowed to cool at room temperature. Finally, the reaction mixture was centrifuged at 5000 rpm for 15 minutes and residue was dried at room temperature.

### 2.4. Characterization Techniques

The formation of metal nanoparticles was confirmed by monitoring the reaction mixture at regular intervals (5 min, 10 min, 15 min, 30 min, 1-hour, 2-hour, 4-hour, and 24-hour for AgNPs and 1-hour, 2-hour, 3-hour, and 4-hour for CuNPs). When the reaction was carried out, during the course of the reaction small amount of aliquot in between some time intervals was withdrawn and dissolved in 3 ml of distilled water and the absorption maximum was scanned by UV-Visible spectra, in a range of wavelength between 200 and 800 nm using UV-Visible Perkin Elmer Lambda 25. Fourier transform infrared (FTIR) spectra of the MNPs were recorded using a Lambda Scientific FTIR-7600 instrument with KBr pellet technique in the range 400–4000 cm^−1^. The particle size, crystal structure, and phase identification of the metal nanoparticles were determined using XRD PW3040/60 X-pert PRO (Netherlands), operating at a voltage of 45 kV and a current of 40 mA with Cu K*α* radiation at 2*θ* angle ranging from 20° to 90°. SEM (Scanning Electron Microscope, JEOL JSM 5600) was used to observe the surface topographies of metal nanoparticles and the elemental analysis was carried out to confirm the presence of metallic silver and copper in the reaction mixture by recording EDX. The morphology and size of the metal nanoparticles were studied by transmission electron microscopy (TEM) analysis using Tecnai 20 G^2^ instrument.

### 2.5. Antibacterial Activity

Synthesized silver and copper nanoparticles were tested for antimicrobial activity by disc diffusion method against pathogenic bacteria* Staphylococcus aureus, Salmonella typhi*,* Escherichia coli*, and* Klebsiella pneumonia *[16]. The sterilized discs were dipped in different concentrations of metal nanoparticles dispersion in dimethyl sulphoxide (DMSO), namely, 100% (10 *μ*g/ml), 75% (7.5 *μ*g/ml), and 50% (5.0 *μ*g/ml), and dried in an oven at 30–40°C. The metal nanoparticles impregnated discs were placed on the plates and kept for incubation at 37°C for 24 hours. After incubation, the different levels of zone of inhibition of bacteria were measured. The standard antibiotic drugs ciprofloxacin and gentamycin were used as reference and 10% DMSO was used as control. The experiments were done in triplicate and mean values of zone diameter were taken.

### 2.6. Haemolytic Activity

In order to know the toxicity of synthesized metal nanoparticles, the hemolysis assay was performed of the silver and copper nanoparticles with acid citrate dextrose (ACD) human blood. Equal weights of silver and copper nanoparticles were taken in different test tubes and 10 mL of phosphate buffer solution was added. Positive and negative controls were produced by adding 0.2 ml of human blood to 4 ml of distilled water and 10 mL PBS solution, respectively. All these test tubes were kept in desiccators for 30 min at 37°C and 0.2 mL of ACD blood was added to each test tube and the test tubes were kept for 1 h in incubator at 37°C. The test tubes were centrifuged at 4500 rpm for 8 min. Optical density (OD) of silver and copper nanoparticles, positive and negative control treated samples, was calculated at 545 nm from 1 mL supernatant [17]

(1) %  of  hemolysis=OD  of  the  sample−OD  of  the  negative  controlOD  of  the  positive  control−OD  of  the  negative  control×100,

where OD of the sample is the optical density of the sample, OD of the negative control is the optical density of the phosphate buffer saline, and OD of the positive control is the optical density of the water.

### 2.7. Antioxidant Activity

The metal nanoparticles were screened for free radical scavenging activity by DPPH method [18]. The AgNPs and CuNPs dispersed in methanol (10–100 *μ*g/ml) were added to different test tubes and the volume was made up to 4 ml using methanol, separately. Then, 3 ml of DPPH (0.1 mM) solution was added and incubated in a dark room for about 30 min at room temperature. All the solution was prepared in the laminar flow. The scavenging activity on the DPPH radical was determined by measuring the absorbance at 517 nm against a blank with an ultraviolet-visible spectrophotometer. Gallic acid and BHT were used as positive control. Tests were performed in triplicate and the results were averaged

(2) %  of  radical  scavenging  activity=  Acontrol−AsampleAcontrol×100,

where *A*_control_ is the absorbance of the control sample (DPPH solution without sample) and *A*_sample_ is the absorbance of the sample (DPPH solution and sample).

### 2.8. Catalytic Activity

The synthesized metal nanoparticles were used as catalyst for the reduction of nitro compounds, that is, 4-nitrophenol and 2-nitrophenol to amino compounds by sodium borohydride [19]. To a 3 ml cuvette containing freshly prepared sodium borohydride (1 ml, 0.2 M) solution, 4-nitrophenol (1.9 ml, 0.2 mM) solution was added. The cuvette was then placed in a UV-Vis spectrophotometer and the absorbance against wavelengths recorded. After adding metal nanoparticles (0.1 ml, 0.1%) solution, the cuvette was shaken vigorously for mixing and kept in a UV-Vis spectrophotometer and scanned from 200 to 800 nm ranges.

## 3. Results and Discussion

### 3.1. UV-VIS Spectroscopic Analysis

**Figure 1:**
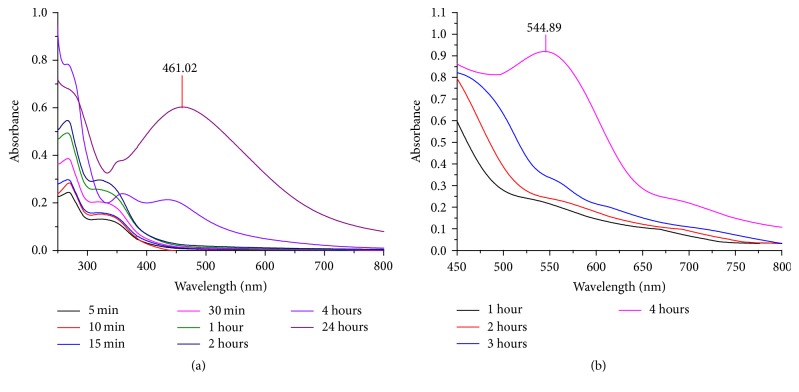
UV-Vis absorption spectra of synthesized (a) AgNPs and (b) CuNPs.

The UV-Vis absorption spectra of AgNPs and CuNPs prepared from leaves extract of* C. occidentalis *are shown in Figure 1. The absorption band of AgNPs occurs at 461.02 nm. The AgNPs exhibit a yellowish-brown color in aqueous solution due to the excitation in UV-visible spectrum depending upon the particle size [20]. The absorption band of CuNPs occurs at 544.89 nm. The absorption bands for CuNPs have been reported to be in the range of 500–600 nm [21]. The absorption peak ascribed to the SPR of Cu particles formed here [22]. The intensity of peak increases as a function of time in both the cases.

### 3.2. FTIR Analysis

**Figure 2:**
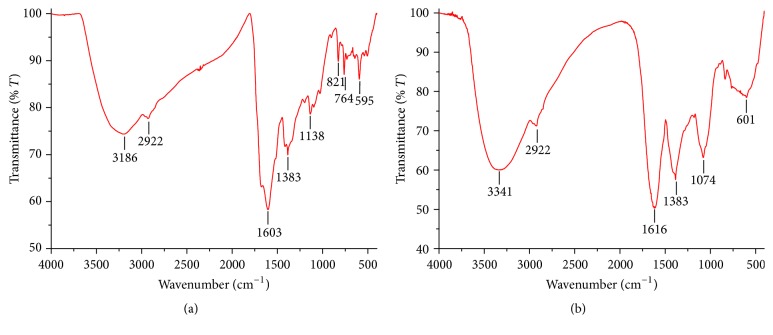
FTIR spectra of synthesized (a) AgNPs and (b) CuNPs.

**Figure 3:**
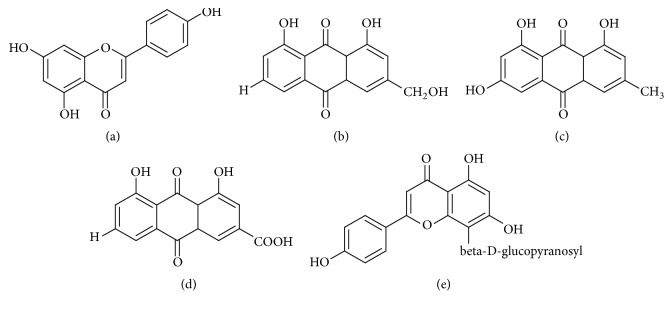
Structure of (a) apigenin, (b) emodin, (c) aloe-emodin, (d) rhein, and (e) vitexin.

The FTIR spectra were carried out to identify the possible biomolecules responsible for capping and reducing agent for the formation of metal nanoparticles. In Figure 2, FTIR spectra of silver and copper nanoparticles show strong absorption band at 1603 cm^−1^ and 1616 cm^−1^, respectively, and it is attributed to binding of NHC=O to metal ions. Other peaks include 2922 cm^−1^ (secondary amine), 1383 cm^−1^ (C-N stretching vibration of aromatic amine), 1138 cm^−1^, 821 cm^−1^, 764 cm^−1^, and 595 cm^−1^ for silver nanoparticles and 1383 cm^−1^, 1074 cm^−1^, and 601 cm^−1^ for copper nanoparticles. The presence of peak at 3186 cm^−1^ and 3341 cm^−1^ could be due to O-H group in polyphenols or proteins or polysaccharide [23, 24]. It has been reported that proteins can bind to metal nanoparticles through the free amine groups or carboxylate ion of amino acid residues. The phytochemical analysis of* Cassia occidentalis *has indicated the presence of phenolic compounds such as apigenin, emodin, aloe-emodin, rhein, and vitexin, which are responsible for the formation of metal nanoparticles (Figure 3) [11].

### 3.3. X-Ray Diffraction Analysis

**Figure 4:**
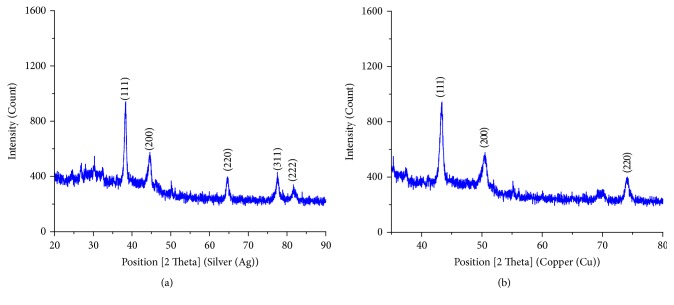
XRD spectrum of synthesized (a) AgNPs (b) CuNPs.

The powder XRD of the metal nanoparticles is recorded between 2*θ* values 20° and 90° and exhibits crystalline nature and is consistent with earlier reports showing possible peaks of silver and copper metal in Figure 4 [15, 25]. Bragg's diffraction peaks for silver nanoparticles are observed at 38.38°, 44.48°, 64.66°, 77.56°, and 81.66° corresponding to 111, 200, 220, 311, and 222, respectively, representing face centered cubic structure of silver. Bragg's reflections for copper nanoparticles are observed in XRD pattern with value of 43.6°, 50.7°, and 74.45° representing 111, 200, and 220 planes of FCC structure of copper. The average crystallite size of AgNPs and CuNPs was calculated to be about 31 nm and 26 nm, respectively, using Scherer formula, *D* = 0.94 *λ*/*β* cos⁡*θ*, where *λ* is incident X-ray wavelength (Cu K*α* = 1.542 Å), *β* is full width half maximum in radians of the prominent line, that is, (111), and *θ* is position of that line in the pattern.

#### 3.3.1. Peak Indexing

The miller indices (*h*  *k*  *l*) were calculated using *d*-spacing values and each peak of the powder diffraction pattern is assigned [26]. The data are given in Tables 1 and 2.

#### 3.3.2. Crystallite Size Calculation

The average crystallite size has been calculated by using Debye-Scherrer formula [27, 28]. The calculated crystallite size details are in Tables 3 and 4.

### 3.4. SEM Analysis

**Figure 5:**
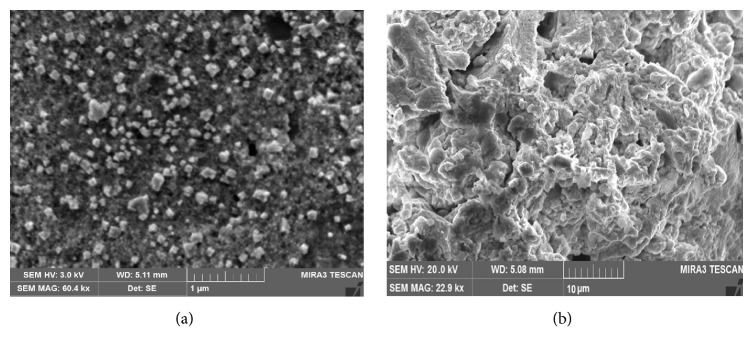
SEM micrograph of synthesized (a) AgNPs and (b) CuNPs.

The surface morphological and nanostructural studies using SEM are shown in Figure 5. The SEM micrographs clearly show well aggregates of silver nanoparticles of* C. occidentalis* with average particle sizes ranging from 20 to 65 nm and copper nanoparticles with 30–65 nm in size.

### 3.5. EDX Analysis

**Figure 6:**
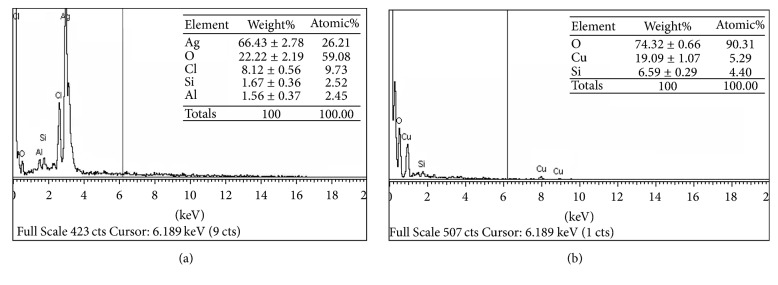
EDX spectrum of synthesized (a) AgNPs and (b) CuNPs.

Figure 6 shows the elemental profile of synthesized silver and copper nanoparticles using leaves extract of* C. occidentalis.* The EDX analysis of silver nanoparticles shows an intense signal at 3 keV indicating the presence of elemental silver in examined samples. The elemental analysis of the silver nanoparticles shown in the figure revealed a strong silver signal (66.43%) along with weak signals of O (22.22%) and Cl (8.12%). Furthermore, two small peaks of Si (1.67%) and Al (1.56%) were also observed in the examined sample. The EDX analysis of copper nanoparticles possesses metallic copper (19.09%) with some other impurities, that is, O (74.32%) and Si (6.59%). This includes elemental peaks at 1.00, 8.00, and 9.00 keV for copper.

### 3.6. TEM Analysis

**Figure 7:**
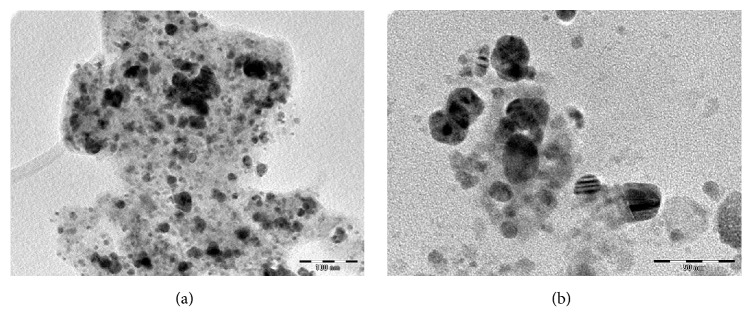
TEM micrograph of the AgNPs using* C. occidentalis *at the scale bar corresponds to (a) 100 nm and (b) 50 nm.

**Figure 8:**
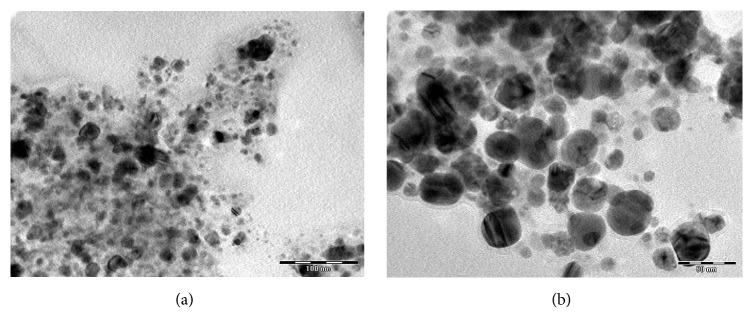
TEM micrograph of the CuNPs using* C. occidentalis *at the scale bar corresponds to (a) 100 nm and (b) 50 nm.

**Figure 9:**
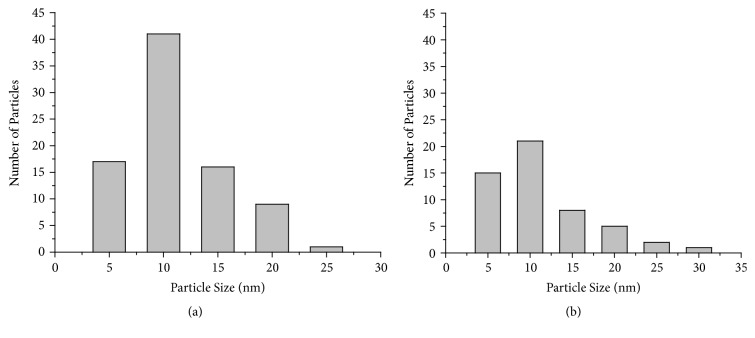
Particle size histogram of (a) AgNPs and (b) CuNPs at the scale bar corresponds to 100 nm.

TEM micrograph shown in Figures 7 and 8 revealed that the particles are spherical, oval, and well dispersed. The particle size of synthesized AgNPs and CuNPs is in the range of 5–25 nm and 5–30 nm, respectively.  Figure 9 shows the histogram of AgNPs and CuNPs of* C. occidentalis*, respectively. It is evident that there is variation in particle sizes.

### 3.7. Antibacterial Activity

Recently, silver and copper nanoparticles were found to be biocidal and as an antimicrobial coating on consumer products [29]. Research reports were available for using silver nanoparticles as antimicrobial agent against Gram positive strains* (S. aureus* and* S. epidermidis)* and Gram negative strains* (E. coli*,* S. typhi*,* Pseudomonas aeruginosa*,* K. pneumoniae*, and* Proteus vulgaris)* [30, 31]. Antimicrobial activity of AgNPs, CuNPs, and standards was tested by disc diffusion method against pathogenic bacteria. Both the particles have similar solubility and were soluble in dimethyl sulphoxide (control) and the zone of inhibition for control was 0 (mm). The activity of MNPs was concentration dependent against tested human pathogens. Antimicrobial activities increased with higher concentration of MNPs, 50% < 75% < 100%. The zone of inhibition increases with increasing the concentration of MNPs [32].  Table 5 summarizes the antibacterial activity of MNPs. The biocapped AgNPs displayed higher antibacterial activity against* E. coli* and CuNPs were more effective against* S. typhi*.

### 3.8. Haemolytic Activity

The haemolytic activity of the silver and copper nanoparticles from* C. occidentalis* was tested against normal human erythrocytes. Haemolytic activity of the MNPs is expressed in percentage haemolysis and reported as mean ± standard deviation of three replicates (Table 6). The toxicity of metal nanoparticles was checked by haemolysis. Haemolysis takes place when the red blood cells come in contact with water and it is important to check this implant material before use. It has been well documented that the permissible limit of haemolysis for biomedical materials should be less than 5% in all the cases [33]. Haemolysis percentage of ACD blood with silver and copper nanoparticles was 1.7% and 4.6%, respectively. Interestingly, silver nanoparticles show even lower haemolysis (1.7%) exhibiting lesser toxicity as compared to copper nanoparticles.

### 3.9. Antioxidant Property

**Figure 10:**
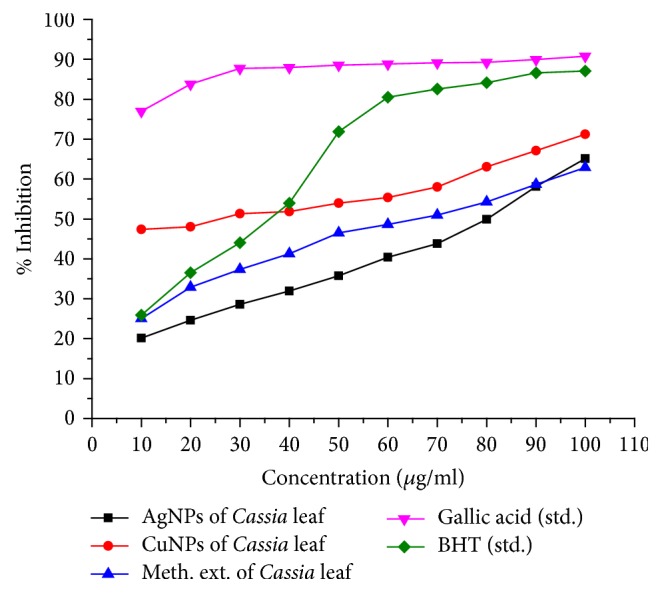
Antioxidant property of AgNPs and CuNPs using leaves extract of* C. occidentalis*.

DPPH is a stable free radical scavenger and shows a characteristic absorption at 517 nm wavelength and after reduction color changes from violet to yellow [34]. The antioxidants react with DPPH and convert it to 1,1-diphenyl-2-picryl hydrazine with decolorisation. The CuNPs showed higher free radical scavenging power than AgNPs and plant extract (Figure 10). The free radical scavenging activity of AgNPs at higher concentration (100 *μ*g/ml) was found to be higher than plant extract. It was found that the IC_50_ value for AgNPs, CuNPs, and plant extract was 69.10 *μ*g/ml, 28.99 *μ*g/ml, and 65.94 *μ*g/ml, respectively. This is due to the efficient oxidation of CuNPs (Cu^0^ + DPPH → Cu^2+^ + 1,1-diphenyl-2-picryl hydrazine). The CuNPs quenched the activity of DPPH by donating copper's electrons.

### 3.10. Catalytic Activity

**Figure 11:**
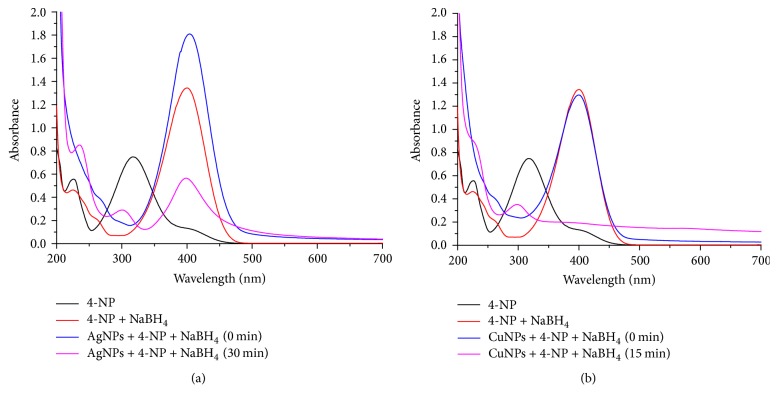
UV-Vis spectrum of the reduction of 4-NP by (a) AgNPs and (b) CuNPs of* C. occidentalis*.

The reduction of 4-nitrophenol (4-NP) and 2-nitrophenol (2-NP) was studied using NaBH_4_ in the presence of synthesized AgNPs and CuNPs using* C. occidentalis* at room temperature and monitored by UV-Visible spectroscopy [19, 35]. The reduction of 4-NP to 4-aminophenol (4-AP) using aqueous NaBH_4_ is thermodynamically favorable (E_0_ for 4-NP/4-AP = 0.76 V and H_3_BO_3_/BH_4_^−^ = 1.33 V versus NHE), but the presence of the kinetic barrier due to large potential difference between donor and acceptor molecules decreases the feasibility of this reaction. The MNPs catalyze this reaction by facilitating electron relay from the donor BH_4_^−^ to acceptor 4-NP to overcome the kinetic barrier. A characteristic absorption peak has been observed for pure 4-NP at 317 nm. The preliminary experiment revealed that the yellow color of 4-NP solution became deeper after the addition of NaBH_4_, and a red shift from 317 to 400 nm occurred due to the formation of 4-nitrophenolate ions [19]. In the absence of any catalyst, the peak at 400 nm remained unchanged even for several days and no reduction of 4-NP was observed. In the presence of AgNPs and CuNPs as a catalyst, the yellow color of 4-NP solution gradually decreased and finally disappeared. In Figure 11, the UV-Vis absorption spectra revealed that the addition of AgNPs induced the lowering of peak intensity at 400 nm and the appearance of a new absorption peak at 293 nm indicating the formation of 4-aminophenol. Finally, the formation of 4-AP is confirmed by the UV-Visible spectrum of pure 4-AP, which shows similar absorption peaks to that of an authentic sample. The complete disappearance of the 400 nm peak was observed within 30 minutes. In case of CuNPs, peak at 400 nm completely disappeared and a new peak was observed at 298 nm within 15 minutes. As compared to AgNPs, the CuNPs have better catalytic ability for the reduction of 4-NP. The rate constants of 4-NP were found to be 4.76 × 10^−2^ min^−1^ with AgNPs and 7.5 × 10^−2^ min^−1^ with CuNPs. Therefore, it is concluded that the CuNPs catalyze the reaction more than the AgNPs.

**Figure 12:**
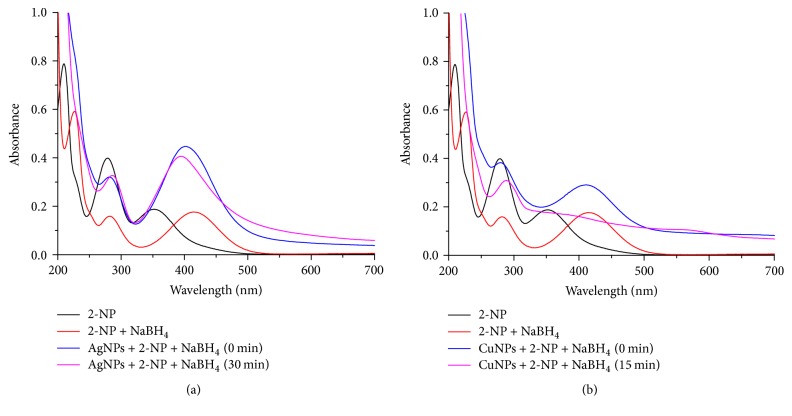
UV-Vis spectrum of the reduction of 2-NP by (a) AgNPs and (b) CuNPs of* C. occidentalis*.

In case of 2-NP, the strong UV-Vis peaks appear at 352 nm and 279 nm. The yellow color of 2-NP was discharged during the course of reduction under identical condition after the addition of NaBH_4_. It was found that there was a very slow decrease of absorbance during the chemical reaction without catalyst. However, after the addition of AgNPs, a decrease in the absorbance at 416 nm and shift of the peak from 279 nm to 285 nm with reaction time usually indicate steady reduction of 2-NP to 2-AP within 30 minutes (Figure 12). The CuNPs reduce 2-NP to 2-AP by sodium borohydride within 15 minutes. The kinetics of this reaction can be followed quantitatively by monitoring the change in the intensity of the peak at 400 nm with time. The reaction was carried out in excess concentration of NaBH_4_ as compared with that of nitrophenols (4-NP and 2-NP) and MNPs so the rate constant can be assumed to be independent of NaBH_4_ concentration. Hence, the reaction is considered as first-order kinetics with respect to nitrophenols concentration. The kinetics of the reaction can be represented using the equation

(3) $$\begin{array}{c} \ln\frac{\left[A\right]}{\left[A_{o}\right]}=-kt, \end{array}$$

where *k* is first-order rate constant, *t* is the reaction time, [*A*_o_] is the concentration of nitrophenols at time *t* = 0, and [*A*] is the concentration at time *t*. The value of [*A*] can be obtained from the absorbance of the peak at 400 nm. The rate constants (*k*) can be calculated from the slope of the plot of ln* A/A*_*o*_ versus reaction time (min). The rate constants of 2-NP were found to be 2.44 × 10^−2^ min^−1^ with AgNPs and 2.75 × 10^−2^ min^−1^ with CuNPs.

The para isomer is absorbed at the longest wavelength with largest *ɛ*_max_ due to extended conjugation but ortho isomer is absorbed at the shorter wavelength with reduced *ɛ*_max_ due to intramolecular hydrogen bonding and steric interactions between the ortho substituents. Therefore, the reduction of 2-NP is slower than that of 4-NP.

## 4. Conclusion

A green method for the synthesis of Ag and Cu nanoparticles using leaves extract of* C. occidentalis* has been developed. The leaves of* C. occidentalis* are capable of producing silver and copper nanoparticles. The metal nanoparticles were characterized by UV-Vis, SEM- EDX, TEM, and XRD measurements. The formation of MNPs was observed by visible color change from yellow to brown and confirmed by UV-Visible analysis. Crystalline nature of the nanoparticles is evident from sharp peaks in the XRD pattern. The biocapped AgNPs displayed higher antibacterial activity against* E. coli* and CuNPs more effectively against* S. typhi*. The CuNPs showed higher scavenging activity than AgNPs. The CuNPs have better catalytic ability for the reduction of 4-NP and 2-NP than the AgNPs. The bioreduction process of the MNPs is an economic and ecofriendly simple one-step method. Green synthesized MNPs have various applications in biochemical-pharmacological investigations such as antimicrobial, antioxidant, anticancer, and wound healing activities. In addition, this green synthetic protocol would be a better alternative to the existing methods.

## Figures and Tables

**Table 1:** Peak indexing from *d*-spacing for AgNPs using *C. occidentalis*.

SI number	2*θ*	*d*-spacing	1000/*d*^2^	(1000/*d*^2^)/60.04	*Hkl*
1.	38.381	2.34	182.62	3	111
2.	44.481	2.03	242.66	4	200
3.	64.664	1.44	482.22	8	220
4.	77.561	1.22	671.86	11	311
5.	81.665	1.17	730.51	12	222

**Table 2:** Peak indexing from *d*-spacing for CuNPs using *C. occidentalis*.

SI number	2*θ*	*d*-spacing	1000/*d*^2^	(1000/*d*^2^)/74.11	*Hkl*
1.	43.389	2.08	231.13	3	111
2.	50.352	1.81	305.24	4	200
3.	74.115	1.27	620.00	8	220

**Table 3:** The crystallite size of AgNPs synthesized using *C. occidentalis*.

SI number	2*θ* value (degree)	FWHM (degree)	Crystallite size (nm)
1.	38.381	0.5936	25.83
2.	44.481	0.7204	21.71
3.	64.664	0.4891	35.04
4.	77.561	0.6183	30.04
5.	81.665	0.4361	43.88

**Table 4:** The crystallite size of CuNPs synthesized using *C. occidentalis*.

SI number	2*θ* value (degree)	FWHM (degree)	Crystallite size (nm)
1.	43.389	0.5839	26.69
2.	50.524	0.8998	17.79
3.	74.115	0.7767	23.36

**Table 5:** Antibacterial activity of AgNPs and CuNPs of *C. occidentalis.*

Bacteria	Zone of inhibition (mm)							
	AgNPs			CuNPs			Ciprofloxacin (std.)	Gentamycin (std.)
	100%	75%	50%	100%	75%	50%		
*S. aureus*	10 ± 0.5	6 ± 0.5	0	10 ± 0.5	5 ± 0.5	0	15 ± 0.5	19 ± 0.5
*E. coli*	13 ± 0.5	12 ± 0.5	10 ± 0.5	11 ± 0.5	8 ± 0.5	5 ± 0.2	20 ± 0.5	20 ± 0.5
*S. typhi*	10 ± 0.6	9 ± 0.2	7 ± 0.5	12 ± 0.5	11 ± 0.5	0	21 ± 0.5	18 ± 0.5
*K. pneumonia*	11 ± 0.5	10 ± 0.2	0	11 ± 0.5	9 ± 0.5	7 ± 0.5	19 ± 0.5	21 ± 0.5

**Table 6:** Haemolytic activity of synthesized silver and copper nanoparticles.

SI number	Metal nanoparticles (MNPs)	Optical density (OD)	Haemolysis percentage (%)
1.	AgNPs	0.078 ± 0.001	1.7%
2.	CuNPs	0.130 ± 0.005	4.6%
3.	Negative sample	0.048	
4.	Positive sample	1.836	
